# Home-based virtual reality-enhanced upper limb training system in children with brain injury: a randomized controlled trial

**DOI:** 10.3389/fped.2023.1131573

**Published:** 2023-05-18

**Authors:** Ja Young Choi, Sook-hee Yi, Dain Shim, Beomki Yoo, Eun Sook Park, Dong-wook Rha

**Affiliations:** ^1^ Department of Physical and Rehabilitation Medicine, Chungnam National University College of Medicine, Daejeon, Republic of Korea; ^2^ Department of Physical and Rehabilitation Medicine, Seoul Rehabilitation Hospital, Seoul, Republic of Korea; ^3^ Department and Research Institute of Rehabilitation Medicine, Yonsei University College of Medicine, Seoul, Republic of Korea

**Keywords:** VR rehabilitation, upper limb function, children with brain injury, home-based rehabilitation, cerebral palsy

## Abstract

**Background:**

Rehabilitation of upper limb function can be challenging in children with brain lesion. Recent virtual reality (VR) rehabilitation may be an additional treatment option in pediatric rehabilitation.

**Objectives:**

To assess the feasibility and effectiveness of a home-based VR-enhanced rehabilitation program with wearable multi-inertial measurement unit (IMU) sensors on upper limb functions in children with brain injury.

**Methods:**

This multicenter single blind randomized controlled trial included 40 children with cerebral palsy (CP) or static brain injury. Subjects were randomized 1:1 to experimental and control group. Both the groups maintained the same therapeutic content and dose of occupational therapy during the intervention period. The experimental group performed additional training at home using the VR-enhanced program for at least 30 min/day, 5 days/week, for 6 weeks. VR training consisted of daily activities or games promoting wrist and forearm articular movements using wearable IMU sensors. The Melbourne Assessment of Unilateral Upper Limb Function-version 2 (MA2), Upper Limb Physician's Rating Scale (ULPRS), Pediatric Evaluation of Disability Inventory-computer adaptive test (PEDI-CAT), computerized 3D motion analysis, and user satisfaction survey were performed. Mann–Whitney U test was used to compare treatment effects between groups, and Friedman and Wilcoxon signed-rank tests were used to compare pre and post intervention.

**Results:**

Overall 35 children (15 in VR group and 20 in control group) completed the protocol. In the experimental group, an average VR training time was 855 min. The accuracy of motion measured by MA2, segmental movements by ULPRS, daily living capability and social cognitive function by PEDI-CAT, movement time and shoulder movement pattern by motion analysis showed significant improvements. However, there were no significant differences in any of the functional outcome measures compared to the control group. All the children and parents reported positive experiences.

**Conclusions:**

Home-based VR training though it had limited impact on improving upper limb function, it could help improve social cognitive function, movement pattern, and efficiency in children with brain injury and could be an effective means of extending clinical therapy to the home.

**Clinical Trial Registration:**

CRIS.nih.go.kr: identifier KCT0003172.

## 1. Introduction

Children with brain injuries, including cerebral palsy (CP), often experience upper limb (UL) spasticity, weakness, and loss of motor coordination, leading to the non-use of their UL in daily living activities. These patients display a form of “developmental disregard,” leads to a further reduction in using the affected UL during functional tasks in daily life (1). Rehabilitation aims to increase the patients' independence and participation in everyday life. Goal-directed and task-specific high-intensity training are key the components of an effective rehabilitation (2).

Home-based programs can be an additional treatment option for patients with motor disorders who may have difficulty maintaining sufficient center-based therapies. Home-based programs using goal-directed training are known to be effective in improving the motor and functional outcomes in children with disabilities (2, 3). Previous systematic review showed that the predominantly used treatment approach in home-based rehabilitation for children with disability is either the constraint-induced movement therapy (CIMT) (33%) or computer-based programs, including virtual reality (VR)-enhanced training (23%) (3). In neurorehabilitation, VR is applied in the aspects of offering repetitive, intensive training with sensory-motor feedback (4). Therefore, home-based VR-enhanced training can provide an environment that may motivate children to practice more frequently and elicit motor learning (5, 6).

Despite the clinical need for home-based interventions, there is a paucity of evidence on home-based VR rehabilitation. There have been only two VR-enhanced home-based randomized controlled trials (RCTs) in children with CP that focused on UL function (7, 8). Additionally, to our knowledge, no study has focused on the distal ULs, including the wrist and hand. In particular, wrist flexion with pronation was one of the leading causes of functional disability in children with CP (9). Therefore, high-quality RCTs are needed to delineate the efficacy of home-based VR training in children with UL dysfunction targeting distal UL function.

Recently, an RCT was conducted in 78 children with UL dysfunction, which showed that 4 weeks of clinic-based VR training improved dexterity functions, the performance of activities of daily living, and forearm supination by kinematic analysis (10). Based on these results, we plan to further investigate whether home based training also can benefit children who cannot attend adequate center-based training. The aims of this study are: (1) to determine the feasibility of using the VR platform in a home-based rehabilitation program and (2) to analyze the effect of home-based VR training in improving UL function in children with brain lesions. The device used in this trial, developed for rehabilitation purposes, utilizes inertial measurement unit (IMU) sensors for real-time feedback and outcome tracking of wrist and forearm articular movements, as well as intensive, task-oriented, repetitive training using various interesting games and functional tasks.

## 2. Materials and methods

### 2.1. Study design

This randomized, controlled, single-blinded, multicenter trial was conducted at three rehabilitation institutions. The internal review board of each participating hospital approved the study, and all parents and patients were informed about the purpose and protocol before enrollment; therefore, written informed consent was obtained. This trial has been registered with the Clinical Research Information Service [identifier: KCT 0003172].

**Figure 1:**
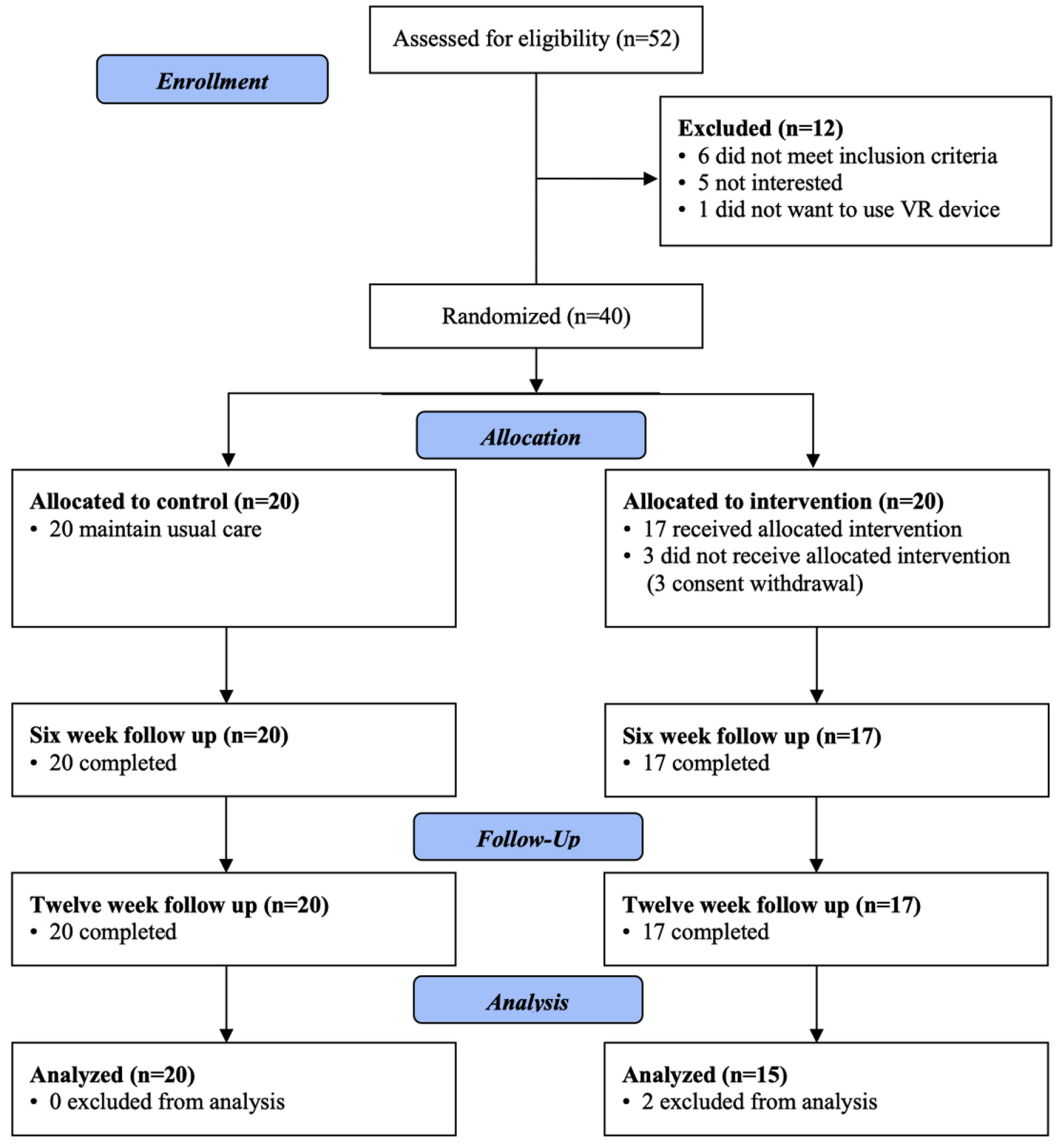
Flow of the participants in the study.

After the baseline assessment, participants were randomized into either the intervention or control group using a centralized web-based randomization system. The system randomly assigned patients to either the experimental or control group in a 1:1 ratio (Figure 1). A randomization sequence was generated at the start of the trial using a computerized R program (version 3.5.1. software).

### 2.2. Participants

The study included children with CP or other static brain lesions, aged 4–17 years, at least 12 months after onset. Children with UL dysfunction who at least actively used their arm with manual ability classification system (MACS) levels II–IV and a house functional classification (HFC) level of 4–7 for the study limb were included. MACS assesses bilateral UL function, whereas HFC assesses unilateral UL function.

Exclusion criteria were children with a history of severe intellectual disability or visual impairment that would interfere with gameplay, botulinum toxin injection, or orthopedic surgery in the UL in the past 6 months. Chemo-denervation, CIMT, surgery, or alternation of anti-spastic medication regimens were prohibited during the 12-week study duration. Forty eligible children were enrolled [mean age: 7.5 (2.8) years]. Thirty-seven children had CP, three had pediatric stroke (age at onset: 3–5 years).

### 2.3. Interventions

Subjects in the control group (*n* = 20) maintained conventional Occupational therapy (OT) without additional intervention. The experimental group (*n* = 20) was guided to perform additional UL training at home using the VR rehabilitation program with wearable IMU sensors for at least 30 min/day excluding set-up time, 5 days/week, for 6 weeks. Both groups maintained their usual care, including conventional OT for the ULs. The amount of conventional OT for both groups was not statistically different during the intervention period (136.2 (95.2) min/week for the intervention group; 108.0 (94.5) min/week for the control group; *p* = 0.214). Both the groups maintained the same therapeutic content and dose of OT during the intervention period.

**Figure 2:**
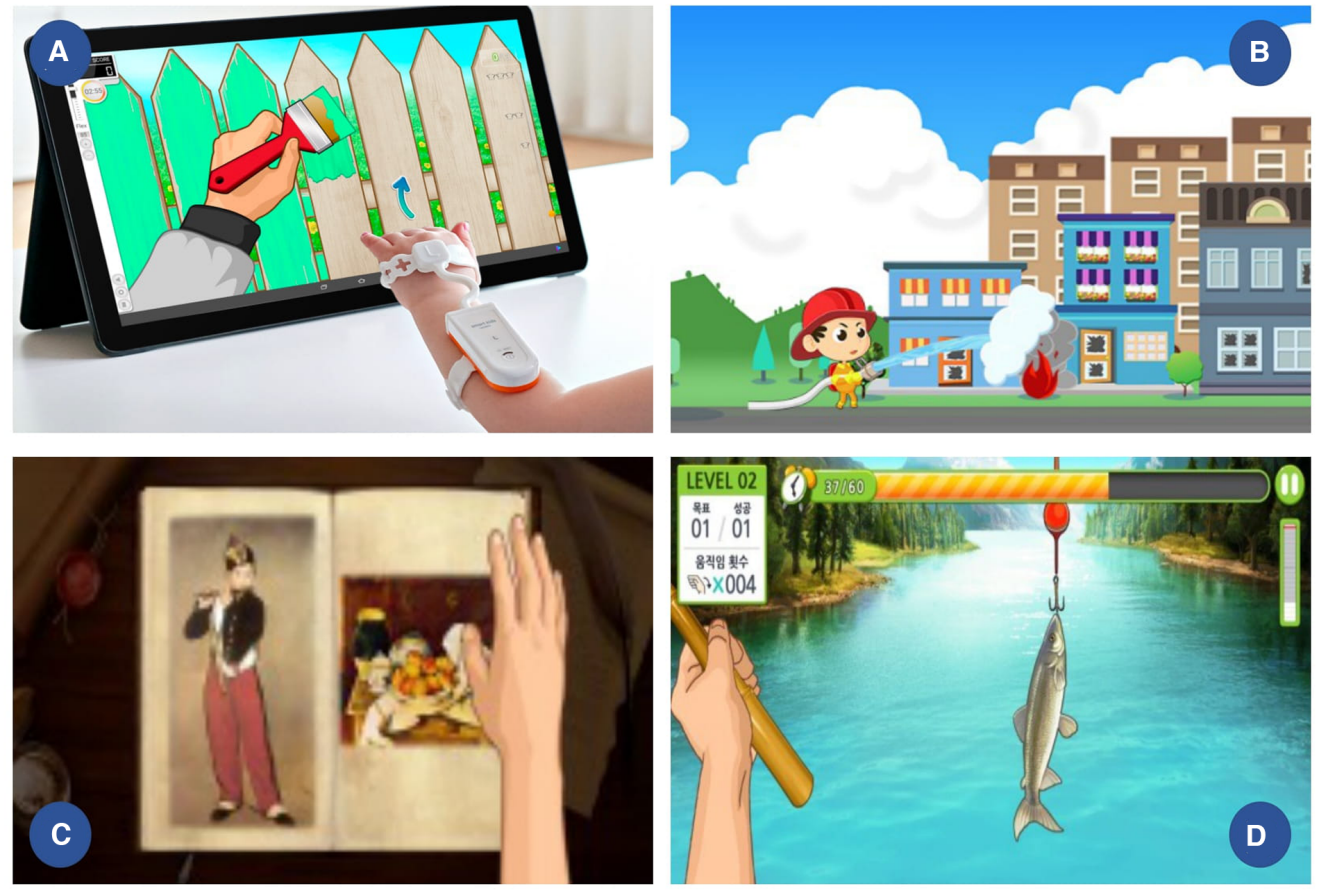
VR device and training contents (**A**) band like wrist attachment device with 2 IMU sensors on hand dorsum and distal forearm; examples of (**B**) wrist extension and flexion (**C**) forearm supination and pronation (**D**) wrist ulnar and radial deviation training contents.

The home-based VR training device used in the experimental group was RAPAEL Smart Kids (Neofect Co. Ltd) (Figure 2). It consists of a band-like wrist attachment device with two IMU sensors—on the dorsum of the hand and distal forearm—and a combined software set-top unit that receives and broadcasts signals to and from the screen. When children move their ULs with the wearable device, the avatar arm on the screen moves in the same way. The VR rehabilitation program comprises several games and simulations including daily activities, that facilitate motions, such as wrist flexion/extension, forearm supination/pronation, and ulnar/radial deviation with or without gravity. At the beginning of the training, the UL capability was assessed using a VR device to determine the initial difficulty level. Subsequently, the difficulty level and content of the training programs were adjusted based on the performance parameters according to the built-in algorithm. This algorithm suggests training program such as forearm supination, wrist extension or deviation depending on the child's functional state. The difficulty ranged from 4 to 10 depending on the training contents. Simultaneous feedback was provided on a computer screen with auditory and visual feedback during the training. The parents helped patients wear the device, motivated them, and inhibited them from using the opposite limb through verbal instruction during training. The home training program was remotely monitored, and the participants were motivated by weekly phone calls. The non-dominant and more involved side were selected as the training limb. However, three participants with bilateral UL dysfunction, whose function on the more involved side was HFC level ≤3 (passive assist level), received training for their dominant UL.

### 2.4. Outcome measures

Both functional and kinematic assessments were performed for all participants at baseline (within 72 h before intervention), at the end of the 6-week intervention (within 1 week after intervention, post-test 1), and after the 6-week follow-up (6 ± 1 week after intervention, post-test 2) to investigate the maintenance of effects. To avoid assessment bias, all assessments were completed by an occupational therapists blinded to the assignment of participants. All the children from the three institutions were evaluated by a blinded assessor to diminish inter-rater bias. Adherence to training was evaluated using the training time and log recorded by the device. Additionally, the analysis of training content for each session according to elicited movements was also analyzed (e.g., forearm supination/pronation, wrist flexion/extension, and ulnar/radial deviation with or without gravity).

#### 2.4.1. Melbourne assessment, version 2 (MA2)

The primary outcome measure was based on the UL motor function assessed with the MA2 post-intervention (11). A total of 14 tasks were scored into the following four subscales with a video recording: range of movement, target accuracy, dexterity, and fluency (11). Scores assigned to each subscale were converted into percentage scores using the maximum possible score.

#### 2.4.2. Upper limb physician's rating scale (ULPRS)

The ULPRS is a semi-quantitative assessment designed to assess the movement pattern, focusing on all three levels of the arm, including the palm, forearm, and elbow (12, 13). It determines whether there is an isolated functional impairment, such as restricted forearm supination, wrist in flexion and deviation. The total score for the unilateral study limb ranged from 0 to 25 and was used for the analysis.

#### 2.4.3. Pediatric evaluation of disability inventory computer adaptive test (PEDI-CAT)

The PEDI-CAT measures functional skills in four domains, including daily activities, mobility, social/cognitive, and responsibility (14). It utilizes a computer adaptive platform with 276 items based on the parent or caregiver report. In our study, scaled scores of each domain ranging from 0 to 100 were used for the analysis.

#### 2.4.4. Computerized three-dimensional motion analysis

The task of drinking from a glass in the sitting position, known to have the least variation in performance, was suitable as a standardized task in assessing the impact of the pathology on movement (15). Participants were asked to reach and grasp a cup on the table at their self-selected speed and repeat the task three times for the study limb. During this reach-and-grasp task, 17 surface markers were attached to trace the joint angles of the UL. Motion capture of the UL was performed using a computerized opto-electric motion analysis system (VICON MX-T10 Motion Analysis System; Motion Analysis; Prime 13^e^) to calculate kinematic data (sampling frequency = 100 Hz).

Motion analysis data were segmented into four sequential phases (Supplementary Figure S1): phase 1, from baseline position to arm extension targeting the object; phase 2, flexing arm and targeting self; phase 3, extending arm and targeting table; and phase 4, retracting arm. As a temporospatial data, movement time, and index of curvature were calculated during each phase (16). As for kinematic analysis, arm variable score (AVS) and arm proﬁle score (APS) were calculated from the kinematic data. AVS provides an index of deviation for a single joint angle, whereas APS is a summary index of UL movement pathology. Subsequently, data from all three trials were processed to calculate each parameter, and the mean values were used for analysis.

#### 2.4.5. User satisfaction survey

The participants’ perceptions of the home-based VR exercise program were investigated using a questionnaire developed for the study. Each participant indicated their agreement with 14 statements about effectiveness, enjoyment, motivation, satisfaction, ease, content suitability, and wearing sensation of the VR-enhanced training using a 5-point Likert scale [strongly disagree (1) to strongly agree (5)].

### 2.5. Statistical analysis

To compare the baseline demographic characteristics of the two groups, either the Mann–Whitney, Chi-square, or Fisher's exact tests was used according to the normality of the variables. Since all variables were nonparametric, the Mann–Whitney *U* test was used to compare the extent of improvement between the VR and control groups. The Friedman and Wilcoxon signed-rank tests were used to compare their state pre- and post-intervention in each group. All statistical analyses were performed using the SPSS version 26 (IBM, USA), and statistical significance was set at *p* ≤ 0.05, with the Bonferroni adjustment for multiple comparisons set as *p* ≤ 0.025.

## 3. Results

A total of 40 children were randomized into two groups; three children in the experimental group dropped out of the trial due to consent withdrawal (Figure 1). Seventeen (43%) and 20 (57%) participants were allocated to the VR and control groups, respectively. Two children in the experimental group were excluded from the analysis because of a lack of adequate training time (87 min, 112 min each). Therefore, 15 participants in the intervention group (MACS level II: III: IV = 8 : 3 : 4, boys: girls = 8: 7) and 20 participants in the control group (II: III: IV = 10; 5 ;5, boys: girls = 10: 10) completed the protocol. There were no significant differences in demographic characteristics, including the weekly conventional OT time (Table 1). No safety issues were reported, and none of the experimental groups experienced any side effects during VR training.

**Table 1:** Characteristics of participants.

Characteristic	VR Intervention group (*n* = 15)	Control group (*n* = 20)	*p*-value^†^
Age (years)	8.1 (3.2)	7.3 (2.6)	0.341
Sex			
Male	8 (53%)	10 (50%)	0.738
Female	7 (47%)	10 (50%)	
MACS			
II	8 (53%)	10 (50%)	1.000
III	3 (20%)	5 (25%)	
IV	4 (27%)	5 (25%)	
HFCS (study limb)			
4	3 (20%)	3 (15%)	0.883
5	4 (27%)	4 (20%)	
6	5 (33%)	10 (50%)	
7	3 (20%)	3 (15%)	
Involved side			
Unilateral	9 (60%)	10 (50%)	0.495
Bilateral	6 (40%)	10 (50%)	
Concurrent OT (min/wk)	138.7 (97.0)	108.0 (99.0)	0.214

Values are expressed as number (%) or mean ± standard deviation (range).

MACS, Manual Ability Classification System; HFCS, House Functional Classification System.

^†^ *p*-values were calculated by Mann-Whitney test, Chi-square tests, or Fisher’s exact test.

### 3.1. Compliance with home-based VR training

The total training time excluding set-up time was 854.6 min, with 29.8 repetitions over 6 weeks, and the average training time per session was 28.6 min (Supplementary Table S1). Two participants in the VR group (S16, S17) attended fewer than five trainings because of repeated hospitalization due to poor physical conditions, such as respiratory infection. Therefore, these two participants were excluded from the analysis. As for training contents, wrist flexion/ extension, wrist deviation, and forearm rotation trainings comprised 65%, 19%, and 16% of the total training, respectively.

### 3.2. Functional assessments

The Friedman test revealed that the UL accuracy function measured by MA2 was significantly improved after the intervention in both groups (*p* < 0.05). However, according to Mann-Whitney test, there was no significant difference between the two groups (Table 2).

**Table 2:** Functional outcome measures at baseline, after intervention, and at 6 weeks follow-up.

Variable	Group	T0	T1	T2	Mann-Whitney test			Friedman test	Wilcoxon signed rank test	
					T0	ΔT0-T1	ΔT0-T2	T0-T2	T0-T1	T0-T2
		Mean (SD)	Mean (SD)	Mean (SD)	*p*	*p*	*p*	*p*	*p*	*p*
**Melbourne Assessment-II**										
Range	VR	69.14 (18.14)	70.87 (14.94)	73.33 (16.22)	0.755	0.254	0.099	0.052	0.155	0.045
	Control	70.74 (20.67)	71.11 (18.96)	71.85 (19.32)				0.835	0.858	0.051
Accuracy	VR	83.20 (10.82)	86.40 (9.89)	88.53 (10.46)	0.179	0.730	0.681	0.032*	0.089	0.010^†^
	Control	84.00 (22.21)	87.00 (19.93)	89.80 (17.72)				0.032*	0.086	0.020^†^
Dexterity	VR	56.25 (20.46)	59.58 (19.46)	58.75 (18.72)	0.364	0.908	0.564	0.223	0.176	0.222
	Control	63.54 (17.53)	66.73 (16.05)	67.71 (17.29)				0.127	0.071	0.083
Fluency	VR	60.00 (15.98)	62.54 (17.25)	62.54 (15.26)	0.755	0.347	0.202	0.057	0.188	0.092
	Control	61.19 (19.62)	61.91 (21.41)	61.19 (19.80)				0.759	0.596	0.724
**ULPRS**										
Total score	VR	19.07 (4.96)	20.00 (3.96)	19.80 (4.59)	>0.999	0.564	0.657	0.008*	0.028	0.016^†^
	Control	19.20 (4.82)	19.65 (4.75)	20.10 (4.90)				0.002*	0.078	0.011^†^
**PEDI-CAT**										
Daily activity	VR	51.73 (4.62)	52.47 (4.31)	53.00 (4.87)	0.856	0.730	0.987	0.002*	0.034	0.004^†^
	Control	51.80 (4.63)	52.50 (4.66)	53.00 (4.24)				0.040*	0.054	0.023^†^
Mobility	VR	61.80 (4.51)	62.00 (4.81)	62.27 (4.61)	0.730	0.254	0.149	0.368	0.527	0.121
	Control	60.40 (6.68)	59.80 (7.01)	59.80 (6.32)				0.522	0.225	0.297
Social cognitive	VR	64.60 (5.34)	65.27 (4.38)	66.00 (4.14)	0.254	0.882	0.254	0.011*	0.325	0.015^†^
	Control	66.60 (3.42)	66.85 (3.33)	67.05 (3.05)				0.062	0.132	0.073
Responsibility	VR	45. 93 (5.51)	47.13 (4.07)	47.40 (3.38)	0.542	0.330	0.882	0.353	0.236	0.185
	Control	46.75 (5.14)	46.70 (4.62)	47.60 (4.95)				0.062	0.726	0.119

Values are expressed as mean (standard deviation).

ULPRS, upper limb physician’s rating scale; PEDI-CAT, pediatric evaluation of disability inventory-computer adaptive test; VR, virtual reality.

Mann–Whitney tests were Between groups; Friedman’s Test was within groups for 3 conditions T0, T1, T2; Wilcoxon was the *post hoc* T0–T1 and T0–T2.

T0, pre-treatment; T1, post-treatment; T2, 1-month follow-up; SD, standard deviation; P, *p*-value; *p < 0.05, ^†^*p* < 0.025 (Bonferroni corrected).

As for the ULPRS, which assesses segmental movements in the affected limb, significant improvements were observed in both groups, with no significant differences. According to PEDI-CAT, both groups demonstrated significant improvements in the daily activity domain after the intervention (*p* < 0.01). Additionally, the social cognitive domain improved in the VR group after the intervention, but there were no significant group differences. A post-hoc Wilcoxon-singed rank test showed that these functional improvements were seen 6 weeks after end of intervention.

### 3.3. Computerized three-dimensional motion analysis

As for motion analysis, shoulder flexion/extension AVS showed significant improvement after intervention in only the VR group (*p* = 0.02) (Table 3). Movement time during phase 2 was significantly reduced in the VR group (*p* = 0.019) compared with that in the conventional group (Supplementary Table S2). The curvature index did not show significant group differences.

**Table 3:** Arm variable score and arm profile score before and after intervention.

		VR group			Control group		
		Pre	Post	*p*-value	Pre	Post	*p*-value
Thorax AVS	Tilt	11.30 (6.75)	10.86 (6.72)	0.79	7.53 (4.42)	10.34 (6.10)	0.06
	Obliquity	4.78 (3.40)	4.36 (3.40)	0.57	4.34 (2.45)	5.43 (3.44)	0.23
	Rotation	7.05 (2.69)	8.17 (4.85)	0.44	8.08 (4.02)	6.58 (5.78)	0.23
Shoulder AVS	Fl/Ext	21.93 (8.57)	18.10 (5.22)	0.02*	17.49 (7.52)	19.44 (7.39)	0.43
	Abd/Ad	17.56 (8.56)	18.95 (10.52)	0.65	14.93 (6.92)	14.91 (6.62)	0.74
	Rotation	20.02 (10.21)	20.78 (11.88)	0.87	15.45 (8.46)	17.13 (7.93)	0.49
Elbow AVS	Fl/Ext	30.79 (8.71)	26.58 (8.22)	0.26	23.74 (8.34)	23.50 (13.01)	0.74
Wrist AVS	Fl/Ext	26.52 (12.13)	28.01 (17.86)	0.65	27.51 (13.66)	25.19 (12.04)	0.55
	Deviation	17.05 (10.13)	16.84 (11.83)	0.43	13.50 (5.92)	16.10 (8.34)	0.12
	Rotation	27.56 (10.08)	25.56 (8.38)	0.43	26.72 (9.86)	25.56 (8.25)	0.54
Total APS		21.70 (2.90)	21.17 (4.14)	0.64	18.89 (3.54)	19.34 (3.17)	0.88

Values are expressed as mean (standard deviation).

AVS, arm variable score; APS, arm profile score; Fl/Ext, flexion/extension; Abd/Ad, abduction/adduction; VR, virtual reality.

* *p*-value <0.05 by Wilcoxon signed rank test.

### 3.4. User satisfaction survey

According to the user satisfaction survey, the mean answered score was 4.0 (0.3) for children, and 3.9 (0.3) for caregivers on a 5-point scale. All the children reported a positive experience (Table 4) and felt the training was safe and interesting.

**Table 4:** User satisfaction survey.

Content		Child	Parent or caregiver
Benefit and effects	Helpful for rehabilitation	3.75 (0.50)	4.25 (0.96)
	Various movement available	4.00 (0.82)	3.50 (1.29)
	Muscle strength improvement	4.00 (0.82)	3.75 (1.26)
	Motor coordination improvement	3.75 (1.26)	3.75 (1.26)
Arousing the interest		4.50 (0.58)	4.25 (0.50)
Motivation	Voluntary participation	4.25 (0.50)	3.75 (0.50)
Level of satisfaction		4.00 (0.82)	4.25 (0.96)
Adaptability	Ease of use in home	4.00 (0.82)	4.00 (0.82)
	Ease of installation and operation	4.00 (0.82)	4.25 (0.96)
	Ease of wear and use	3.75 (0.50)	4.25 (0.96)
Contents suitability	Understanding of the task	4.50 (0.58)	3.75 (0.50)
	Contents suitability	–	4.25 (0.50)
	Level of difficulty for child	–	3.50 (1.00)
Wearing Sensation	Product safety	3.50 (1.00)	4.25 (0.96)

Values are expressed as mean (standard deviation).

## 4. Discussion

We report a randomized controlled multicenter trial using a home-based VR training system developed for rehabilitation purposes in children with brain lesions. Participants' compliance with this home-based therapy regimen was high for most and showed no adverse events. In the VR group, the shoulder movement pattern significantly improved, and the movement time was shorter than that in the control group. However, there were no significant group differences in the functional parameters.

Although the VR device in this study focuses on distal training, it can improve the proximal compensation pattern and efficiency of movement, which shortens movement time. During the reaching tasks, the shoulder executed a flexion movement, while the elbow extended toward the target. Children with sensorimotor deficits tend to move their shoulder joints with less complex coordination during reach to grasp action (17). In this study, after 6 weeks of VR training, this proximal abnormal movement pattern was improved, which was confirmed through the improvement of the shoulder AVS value. The movement time reduction reported in this study echoed results from previous research (18), which showed improvements in task execution time after VR training.

Notably, social cognitive function improved only in the VR group, although there was a lack of statistical significance across group differences. The social cognitive domain of the PEDI-CAT is composed of communication, interaction, and everyday cognitive components. Visuospatial perception, eye–hand coordination, and prompt reaction are needed during the training of VR-enhanced content in this study. These abilities might be related to improvements in social cognitive function after VR training. However, further confirmative studies on whether VR training can improve cognition and elucidation of its mechanism are needed.

Home-based rehabilitation programs enable children to incorporate training into their daily routines, offering generalization and repetitive training that enhances motor learning (19). Adherence to traditional home exercise programs is often poor for children with CP (20). Gamification of VR-based rehabilitation programs can motivate children to actively participate in rehabilitation because some boring or repetitive exercises have been substituted by more interactive and enjoyable activities.

Until now, only two RCTs on VR-enhanced home-based UL training have been reported in children with CP (7, 8). One trial using a commercial device did not show significant effectiveness (8). The other trial using a web-camera-based motion detection with a mean of 32.4 h of training led to an improvement in ADL performance (7). However, both studies failed to demonstrate improvements in impaired UL function. The VR training program that focused on the distal UL in this study also showed limited results in functional outcomes, whereas previous center-based trials with occupational therapists conducted by our research team using the same device showed significant improvement with less training time (600 min) (10). In this regard, home-based system should be able to complement dedicated content or some additional coaching and monitoring system to help users focus more on treatment to achieve a therapeutic effect. Home based rehabilitation has generally used two communication approaches, synchronous (real time and interactive) and asynchronous (delayed) communication (21). The asynchronous training used in this study has the advantage of greater flexibility and convenience of scheduling related to high compliance; While the limitation is the lack of direct, real-time supervision and feedback during exercise.

There is solid evidence that the dose of therapy is one of the key factors to attain changes in performance (22). However, there is no consensus on the appropriate training duration, method, and intensity for home-based programs; the duration varies from 2 weeks to 6 months, and the intensity ranges from 70 min to 56 h a week. As for children, they needed to practice for more than 30–40 h to improve general UL function according to a systematic review (23). However, it is believed that lower doses of practice are needed to achieve task specific skills; 14–25 h of combined face-to-face therapy (5.6 h) and home practice (8.4 h), if children have set their own individual goals and have practiced those goals (23). Future dose research could add significant insights into the minimum threshold dose and critical components of interventions. Clinical guidelines for VR-enhanced home programs, including training time and intensity, methods for providing support and coaching, and evaluation of outcomes, are also needed.

Feasibility is an important aspect to consider when implementing home-based programs. Feasibility components, such as satisfaction, implementation, acceptability, or practicality, are important factors in determining whether an intervention is applicable. According to the satisfaction survey in this trial, the interest and motivation scores were 4.5 (0.6) and 4.3 (0.5), respectively. Compliance and adherence are also important feasibility factors for home-based programs. Gaming properties, including immediate audiovisual feedback, challenging tasks, and optimal difficulty, can provide a sense of achievement and enjoyment, thereby motivating active participation in the rehabilitation process, which can lead to better functional outcomes. Additionally, parents play a significant role in home-based programs. They may experience pressure to comply with the program. However, VR enhances home programs that allow children to exercise themselves to some extent, reducing the burden on parents or caregivers. In this context, it is noteworthy that caregivers' satisfaction scores were high. A follow-up study on the change in stress index of parents before and after training is also needed.

Recently, the need for telerehabilitation is increasing to provide medical services anywhere, anytime, even directly at home (24–26). Several studies have reported that game-based telerehabilitation tends to contribute to the improvement of UL motor function in individuals with CP (26, 27). In this regard, VR-enhanced UL training program has the potential for providing UL training through telerehabilitation for children with CP. Additionally, remote VR telerehabilitation strategies for patients at home, which could reduce travel burden and costs, deserve further examination.

The present study has several limitations. Many daily living tasks require bimanual activities. In this regard, bimanual training in the children's daily home environment seems to be the best approach to realizing the performance of acquired skills in the daily living environment. And the device in this trial could not support hand movements due to the difficulty of customizing various finger lengths in children. Future programs for bimanual training and/or including finger motion are needed to improve function. Additionally, the sample size was small for the subgroup analysis. Further larger controlled studies are needed to delineate the factors associated with the effectiveness of home-based training.

In conclusion, this home-based VR training could help improve social cognitive function, shoulder movement pattern, and shorten movement time in children with brain injury; however, in this study, home-based training still had a limited impact on improving UL function. Nevertheless, VR exercises may be an effective means of extending clinical therapy to the home. The feasibility results indicated that the VR training program was both motivational and helpful. This new home-based UL training system using VR could be a promising motivational training tool for children with brain injuries.

## Data Availability

The original contributions presented in the study are included in the article/**Supplementary Material**, further inquiries can be directed to the corresponding author.
