# Supplementary material for: Home-based virtual reality-enhanced upper limb training system in children with brain injury: a randomized controlled trial
Source: Front Pediatr. 2023 May 18;11:1131573. doi: 10.3389/fped.2023.1131573 (PMC10233002; doi:10.3389/fped.2023.1131573)
Supplement: Supplementary file 1 [file Datasheet1.pdf]

Supplementary Material

Supplementary Table 1. Training information

	Training amount during 6wks (min)			Training Movement				
	Number of training sessions	Total training time, min	Mean training time per session, min	Forearm pron/sup (%)	Wrist UD/RD (%)	Wrist UD/RD-EG (%)	Wrist Flex/Ext (%)	Wrist Flex/Ext-EG (%)
Subject01	41	1368	33.4	13.4	10.8	7.5	27.9	40.3
Subject02	37	1175	31.8	13.3	12.3	11.1	25.6	37.6
Subject03	33	990	30.0	12.1	9.8	8.0	37.8	32.3
Subject04	29	978	33.7	15.8	8.6	8.4	31.0	36.2
Subject05	31	973	31.4	15.8	9.5	9.8	31.1	33.8
Subject06	31	930	30.0	12.5	9.7	12.1	23.8	41.9
Subject07	29	884	30.5	16.6	13.8	7.0	23.9	38.7
Subject08	27	863	32.0	8.6	13.1	10.8	30.0	37.6
Subject09	35	850	24.3	15.0	7.8	10.9	25.1	41.1
Subject10	27	803	29.7	12.6	10.1	9.3	34.0	34.0
Subject11	24	744	31.0	16.5	4.6	3.4	64.0	11.5
Subject12	33	679	20.6	17.1	9.4	8.8	30.7	35.0
Subject13	30	627	20.9	30.1	13.1	12.7	23.8	20.3
Subject14	28	618	22.1	24.0	6.3	12.3	21.6	35.8
Subject15	12	337	28.1	18.6	5.5	9.9	34.0	32.0
Mean (1-15)	29.8 (6.5)	854.6 (245.2)	28.6 (4.4)	16.0 (5.2)	9.7 (2.8)	9.5 (2.4)	30.7 (9.7)	33.9 (8.1)
Excluded from analysis								
Subject16	5	112	22.5	14.8	3.4	2.7	33.3	45.9
Subject17	5	87	17.4	34.1	11.5	4.8	24.9	24.9

UD, ulnar deviation; RD, radial deviation; EG, eliminated gravity

Supplementary Table 2. Motion analysis at baseline, after intervention, and at 6 weeks follow-up

Variable	Task phase	Group	T0	T1	T2	Mann-Whitney test			Friedman Test	Wilcoxon signed rank test	
						T0	ΔT0-T1	ΔT0-T2	T0-T2	T0-T1	T0-T2
			mean (SD)	mean (SD)	mean (SD)	P	P	P	P	P	P
**Temporal-spatial Parameter**											
Movement Time	Phase 1	VR	1.46 (0.59)	1.38 (0.56)	1.64 (0.75)	0.382	0.908	0.908	0.819	0.733	0.532
		Control	1.37 (0.68)	1.34 (0.69)	1.55 (0.96)				0.848	0.794	0.478
	Phase 2	VR	1.97 (1.24)	1.97 (1.21)	1.52 (0.84)	0.934	0.961	0.019*	0.138	0.510	0.061
		Control	1.72 (0.80)	1.89 (1.29)	1.98 (1.13)				0.238	0.286	0.263
	Phase 3	VR	1.64 (0.83)	1.57 (0.67)	1.46 (0.86)	0.934	0.705	0.214	0.247	0.733	0.307
		Control	1.73 (1.11)	1.66 (1.30)	1.91 (1.83)				0.387	0.940	0.526
	Phase 4	VR	1.28 (0.58)	1.49 (0.50)	1.70 (0.93)	0.382	0.382	0.438	0.280	0.047	0.078
		Control	1.59 (0.85)	1.66 (1.22)	1.61 (0.70)				0.675	0.896	0.681
Index of Curvature	Phase 1	VR	1.42 (0.30)	1.49 (0.43)	1.34 (0.65)	0.633	0.657	>0.999	0.627	0.776	0.865
		Control	1.40 (0.36)	1.34 (0.22)	1.43 (0.35)				0.861	0.502	0.765
	Phase 2	VR	1.26 (0.21)	1.26 (0.32)	1.11 (0.58)	0.107	0.080	0.542	0.807	0.638	0.427
		Control	1.18 (0.24)	1.21 (0.30)	1.16 (0.10)				0.504	0.079	0.520
	Phase 3	VR	1.30 (0.32)	1.20 (0.10)	1.24 (0.97)	0.587	0.856	0.755	0.420	0.820	0.334
		Control	1.33 (0.53)	1.23 (0.34)	1.20 (0.24)				0.165	0.502	0.108
	Phase 4	VR	1.52 (0.63)	1.49 (0.37)	1.65 (1.13)	0.856	0.831	0.542	0.189	0.733	0.570
		Control	1.61 (0.81)	1.53 (0.47)	1.58 (0.47)				0.387	0.627	0.654

VR, virtual reality; Values are Least square mean (standard error)

* p <0.05 by linear mixed model

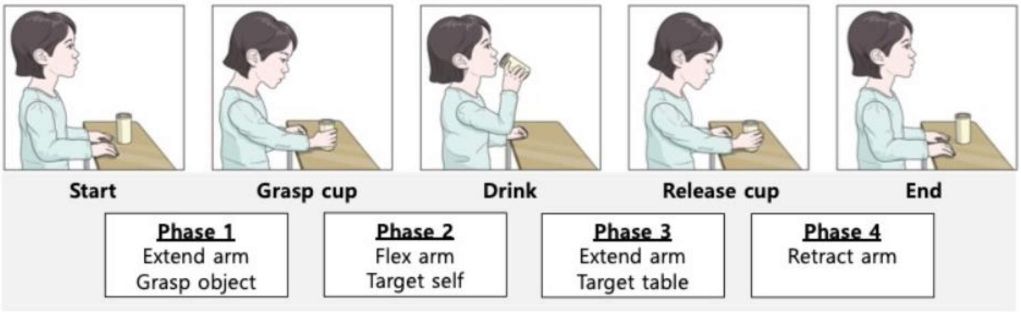

Supplementary Figure 1. Segmentation of motion capture data of the reach-and-grasp task
